# Purification of native histidine-rich protein 2 (nHRP2) from *Plasmodium falciparum* culture supernatant, infected RBCs, and parasite lysate

**DOI:** 10.1186/s12936-021-03946-1

**Published:** 2021-10-17

**Authors:** Balwan Singh, Jessica N. McCaffery, Amy Kong, Yong Ah, Scott Wilson, Sayan Chatterjee, Deepak Tomar, Michael Aidoo, Venkatachalam Udhayakumar, Eric Rogier

**Affiliations:** 1 Malaria Branch, Division of Parasitic Diseases and Malaria, Centers for Disease Control and Prevention, Atlanta, GA 30329 USA; 2 IHRC, Inc, Atlanta, GA 30346 USA; 3 Department of Medicine, Division of Rheumatology, Emory University, Atlanta, GA USA; 4 Lowance Center for Human Immunology, Emory University, Atlanta, GA USA

**Keywords:** *Plasmodium falciparum*, Antigen, Histidine-rich protein 2 (HRP2), Protein purification

## Abstract

**Background:**

Despite the widespread use of histidine-rich protein 2 (HRP2)-based rapid diagnostic tests (RDTs), purified native HRP2 antigen is not standardly used in research applications or assessment of RDTs used in the field.

**Methods:**

This report describes the purification of native HRP2 (nHRP2) from the HB3 *Plasmodium falciparum* culture strain. As this culture strain lacks *pfhrp3* from its genome*,* it is an excellent source of HRP2 protein only and does not produce the closely-related HRP3. The nHRP2 protein was isolated from culture supernatant, infected red blood cells (iRBCs), and whole parasite lysate using nickel-metal chelate chromatography. Biochemical characterization of nHRP2 from HB3 culture was conducted by SDS-PAGE and western blotting, and nHRP2 was assayed by RDT, ELISA, and bead-based immunoassay.

**Results:**

Purified nHRP2 was identified by SDS-PAGE and western blot as a − 60 kDa protein that bound anti-HRP-2 monoclonal antibodies. Mouse anti-HRP2 monoclonal antibody was found to produce high optical density readings between dilutions of 1:100 and 1:3,200 by ELISA with assay signal observed up to a 1:200,000 dilution. nHRP2 yield from HB3 culture by bead-based immunoassay revealed that both culture supernatant and iRBC lysate were practical sources of large quantities of this antigen, producing a total yield of 292.4 µg of nHRP2 from two pooled culture preparations. Assessment of nHRP2 recognition by RDTs revealed that Carestart Pf HRP2 and HRP2/pLDH RDTs detected purified nHRP2 when applied at concentrations between 20.6 and 2060 ng/mL, performing within a log-fold dilution of commercially-available recombinant HRP2. The band intensity observed for the nHRP2 dilutions was equivalent to that observed for *P. falciparum* culture strain dilutions of 3D7 and US06 F Nigeria XII between 12.5 and 1000 parasites/µL.

**Conclusions:**

Purified nHRP2 could be a valuable reagent for laboratory applications as well as assessment of new and existing RDTs prior to their use in clinical settings. These results establish that it is possible to extract microgram quantities of the native HRP2 antigen from HB3 culture and that this purified protein is well recognized by existing monoclonal antibody lines and RDTs.

**Graphical Abstract:**

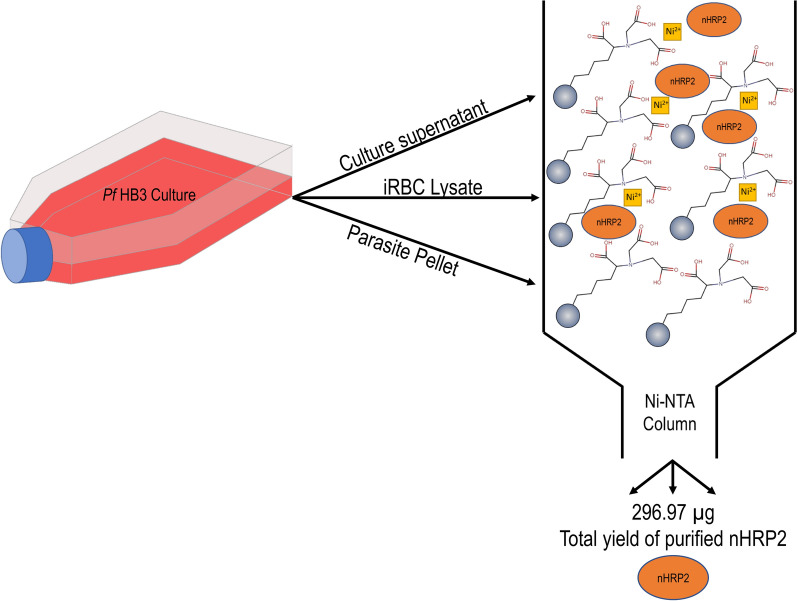

**Supplementary Information:**

The online version contains supplementary material available at 10.1186/s12936-021-03946-1.

## Background

In 2010, the World Health Organization (WHO) recommended confirmation of all suspected malaria infections for better case management and to reduce the unnecessary use of artemisinin-based combination therapy (ACT) and other anti-malarial drugs [1–4]. To meet the need for universal access to testing at all levels of the healthcare system, point-of-care rapid diagnostic tests (RDTs) that detect malaria antigens have become a widespread method for malaria diagnosis in some parts of the world as these tests can be performed by personnel in remote settings with only minimal training required [3, 5].

By far, the most utilized RDT type is that which detects histidine-rich protein 2 (HRP2), a protein produced exclusively by *Plasmodium falciparum* [6, 7]. In 2019, *P. falciparum* was responsible for 99.7% of all malaria infections in sub-Saharan Africa (SSA) [7], and a majority of the 412 million RDTs sold in SSA in 2018 were HRP2-based [8]. In addition to the qualitative detection of HRP2 for diagnosis of active infection by RDTs, determining the concentration of HRP2 in blood samples by other laboratory assays has been used to estimate parasite density [9], attribute febrile illness to malaria [10], and provide estimates on malaria transmission intensity [11, 12].

The HRP2 antigen was first identified in 1974 in the avian parasite *Plasmodium lophurae* [13], and since then, three unique histidine-rich proteins have been identified in the *P. falciparum* genome [8]. These include the knob-associated histidine-rich protein, PfKAHRP (also known as HRP1 [14, 15]), as well as HRP2 and HRP3 [16–18], which were all characterized shortly after the methods for *P. falciparum* culture were first published in 1976 by Trager and Jensen [19]. HRP2 is closely related to HRP3 with 85–90% homology in the nucleotide sequence which includes multiple histidine repeat domains [8]. From different global isolates, the two exons of HRP2 encode for a protein with a range of 600–960 amino acids [20] which includes numerous repeating epitopes composed of approximately 34% histidine, 37% alanine, and 10% aspartic acid [16].

HRP2 is highly stable and abundantly expressed during the erythrocytic stage of *P. falciparum* infection, resulting in accumulation of HRP2 in the erythrocyte cytoplasm and upon rupture of the erythrocyte, its release into host plasma. Due to the high levels of expression of this protein, HRP2 has been found in plasma [21], saliva [22], and urine [23] of persons infected with *P. falciparum.* Furthermore, since treatment of falciparum malaria with ACT removes parasites from infected erythrocytes through splenic pitting and allows for recirculation of previously infected red blood cells, following the killing of erythrocytic stage parasites, HRP2 remains detectable by RDT and other HRP2-based assays for weeks following parasite treatment [8, 24, 25].

Even with the widespread use of HRP2-based RDTs, purified native HRP2 (nHRP2) has not been produced at large quantities for use in research or the assessment of HRP2-RDTs used in the field. Recombinant HRP2 (rHRP2) production can be done on a large scale but has resulted in material with variable reactivity on some RDTs [6], and a user would be limited by only one sequence of rHRP2 encoded by the plasmid. Because of the importance of HRP2 detection in diagnostics and malaria research, it is possible that the use of nHRP2 purified from in vitro culture of *P. falciparum* could play an important role in the quality assurance of field RDTs and the development of improved detection assays for this antigen. This report describes the purification and subsequent biochemical characterization of nHRP2 from the *P. falciparum* culture strain HB3. The nHRP2 protein was isolated from supernatant, infected red blood cells (iRBCs), and whole parasite lysate using nickel-metal chelate chromatography. The purified nHRP2 was assayed by three different detection platforms: RDT, ELISA, and bead-based immunoassay.

## Methods

### Materials collected from *Plasmodium falciparum* HB3 in vitro culture

All procedures were followed in accordance with CDC malaria parasite culture protocols and performed in the biosafety cabinets using aseptic technique. The HB3 strain was selected as it is an established culture strain that has the gene for *pfhrp3* deleted and therefore does not express HRP3 antigen [26], allowing for the purification of HRP2 without risking contamination with the paralogue HRP3. *Plasmodium falciparum* HB3 parasites used in this study had previously been cryopreserved in a 57% glycerolyte solution. Vials were thawed for 1–2 min in a water bath maintained at 37 °C with gentle shaking while ensuring that the cap remained above the water level. To prevent lysis of the RBCs, the 1–2 mL volume of frozen parasite stock was transferred to a 50 mL conical tube for processing with a series of sterile salt solutions of decreasing concentration starting at 12% NaCl. A volume of 0.2 mL of 12% NaCl per mL of thawed culture stock was added drop by drop to the conical tube while placed on a low-rpm vortex. Following the addition of the first salt solution, the culture sample was incubated for 3 min without shaking, then 10 mL of 1.6% NaCl was added per mL of culture as before. The parasite-salt solution was then centrifuged at 500×*g* for 10 min and the supernatant was removed by aspiration to leave 0.5 mL. To each culture stock, 10 mL of a 0.9% NaCl + 0.2% dextrose solution was added per mL of initial culture stock as before and the sample was centrifuged. The supernatant was then removed to leave 0.5 mL volume containing a packed pellet of parasites for inoculation.

The parasite pellet was then transferred to a T25 culture flask containing 4.5 mL of complete medium consisting of RPMI 1640 (Gibco, ThermoFisher Scientific, Waltham, MA, USA) with 10% human O + serum, prewarmed to 37 °C, by first resuspending the pellet in the 50 mL conical tube using 2 mL of warmed media from the flask. Washed human O + erythrocytes were added to the flask to obtain a total RBC volume ≤ 0.5 mL and mixed by recapping and gently shaking. The culture flask was then gassed with a mixture of 5% CO_2_ + 5% O_2_ and 90% N_2_ for 30–45 s and then tightly capped and placed on an orbital rotator in a 37 °C incubator without CO_2_. Parasite cultures were maintained daily by changing out the medium with complete RPMI prewarmed to 37 °C. The old culture media was collected each day and labelled with the parasitaemia and stored at − 20 °C for further use. Following the addition of new media, the flask was gassed with the CO_2_ + O_2_ + N_2_ mixture as before and placed back on an orbital rotator inside the 37 °C incubator. Parasitaemia values were obtained daily from thin blood smear readings using Giemsa stain. To prepare a thin blood smear from the culture flask, a 200 µL aliquot of the culture was transferred to a 1.5 mL Eppendorf tube and centrifuged. Most of the supernatant was then removed, leaving only a volume of supernatant equal to that of the pellet for the thin smear. The culture flask was subcultured every third day, or when the parasitaemia was greater than 3%, to an increasing flask size, starting at a T25 flask and going to a T75 flask then a T150 flask. The larger flask size allowed for a larger total volume of the resulting sample. Upon each subculture, new cultures were started with at least 1% parasitaemia at 10% haematocrit. The flasks containing the subcultured sample were then placed onto an orbital rotator inside the incubator overnight on a daily basis to increase the overall number of single-infected iRBCs. This process allowed the parasitaemia to increase exponentially within a few days, especially when there were many schizonts present. The supernatant was harvested daily by transferring the content of each culture flask into a sterile centrifuge tube, centrifuged at 500×*g* for 10 min to separate the supernatant from the pellets. The supernatant was then pipetted into another sterile centrifuge tube, labelled with the strain name, date and parasitaemia, and stored at − 20 °C for later use. Cultures were harvested when parasitaemia was between 9 and 12%, usually around the fourth day. If the parasitaemia of the cultures was less than 9% after the fourth day since subculture, the culture was subcultured to a new flask or flasks, at a starting parasitaemia of 1% and at 10% haematocrit.

### Purification of native HRP2 antigen from culture supernatant, iRBCs, and whole parasite lysate

All centrifugations were carried out using a Sorvall RC6 Plus Superspeed centrifuge (Thermo Fisher Scientific, Waltham, MA, USA), using a Sorvall GS3 rotor for centrifugation at 1200 and 1500 rpm and a Sorvall SS-34 rotor for centrifugation at 17,000 rpm. Supernatant pools were centrifuged at 1500 rpm for 10 min before being filtered through a 0.45 µM filter (Corning, Corning, NY, USA), and an equal volume of 4 °C 1X phosphate buffer with 50 mM NaH_2_PO_4_, and 300 mM NaCl at pH 8.0 was added to the pooled supernatant. Imidazole (Sigma, St. Louis, MO, USA) was added to each supernatant pool to obtain a final concentration of 10 mM imidazole and allowed to incubate at 4 °C with continuous stirring at 1500 rpm for 2 h. The mixture was centrifuged at 1200 rpm for 5 min and nHRP2 was purified using affinity chromatography through Ni–NTA slurry using the batch purification method described by the manufacturer (Qiagen, Hilden, Germany). Briefly, 1 mL of Ni–NTA agarose was used for every 250 mL of supernatant and mixed via gentle shaking at room temperature for 1 h. Because the Ni–NTA column methodology relies on the immobilization of histidine-rich targets by nickel (Ni), the use of the HB3 culture strain which has HRP2 but lacks the histidine-rich HRP3 protein is essential to ensure the purification of only HRP2 and not HRP3.

The supernatant-Ni–NTA mixture was loaded into the chromatography column and the flow-through was collected. The Ni–NTA slurry in the column was washed with 20 mM Imidazole, and protein was subsequently eluted using washes with 500 mM Imidazole and collected. Each fraction, including flow-through, washes, and elutes was tested for purity and molecular weight estimation of the antigen present using sodium dodecyl sulfate–polyacrylamide gel electrophoresis (SDS-PAGE) and western blotting followed by assessment via bead-based HRP2 detection assay as described below. In order to estimate the efficiency of nHRP2 binding to the Ni–NTA column, loss of antigen during the washing steps, and absolute elution of antigen from the column, each fraction during the column purification was assessed for levels of nHRP2. Two independent column purification experiments (Exp 1 and Exp 2) were performed under the same wash and elution conditions to purify nHRP2 obtained from the HB3 culture supernatant (Table 1), iRBCs (Table 2), and parasite pellet (Table 3), however the per cent parasitaemia of the starting samples differed between the two experiments.

**Table 1:** Amount of nHRP2 obtained from HB3 culture supernatant from two experiments

Experiment	Sample	Imidazole (mM)	HRP2 conc (ng/mL)	Volume obtained (mL)	Total nHRP2 (µg)
1	Culture supernatant		40.88	3662	149.711
1	Column flow-through		0.89	3662	3.262
1	Wash 1	10	0.07	45	0.003
1	Wash 2	10	0.03	47	0.001
1	Wash 3	20	0.04	50	0.002
1	Wash 4	20	0.04	47	0.002
1	Wash 5	50	0.28	9	0.003
1	Wash 6	50	2.22	9	0.020
1	Elution 1	500	10,542.21	10	105.422
1	Elution 2	500	3548.24	10	35.482
1	Elution 3	1000	1331.44	6.5	8.654
1				**TOTAL µg eluted**	**149.6**
1				**% Recovery**	**99.9**
2	Culture supernatant		9.61	480	4.612
2	Column flow-through		0.11	480	0.054
2	Wash 1	10	0.05	11.5	0.001
2	Wash 2	10	0.01	12	0.000
2	Wash 3	10	0.00	11	0.000
2	Wash 4	10	0.00	12.5	0.000
2	Wash 5	10	0.00	12	0.000
2	Wash 6	10	0.00	12	0.000
2	Wash 7	20	0.01	7.5	0.000
2	Wash 8	20	0.01	13	0.000
2	Wash 9	20	0.01	9	0.000
2	Elution 1	500	3311.53	1.5	4.967
2	Elution 2	500	2926.64	1.5	4.390
2	Elution 3	500	1826.29	1.5	2.739
2	Elution 4	500	705.83	1.5	1.059
2	Elution 5	500	214.17	2	0.428
2				**TOTAL µg eluted**	**13.6**
2				**% Recovery**	**295.7**

**Table 2:** Amount of nHRP2 obtained from HB3 infected RBCs from two experiments

Experiment	Sample	Imidazole (mM)	HRP2 conc (ng/mL)	Volume obtained (mL)	Total nHRP2 (µg)
1	Lysed RBC supernatant		87.58	1800.0	157.652
1	Column flow-through		0.42	1800.0	0.753
1	Wash 1	10	0.04	35.0	0.002
1	Wash 2	10	0.03	40.0	0.001
1	Wash 3	10	0.03	42.0	0.001
1	Wash 4	10	0.02	42.0	0.001
1	Wash 5	20	0.02	32.0	0.001
1	Wash 6	20	0.01	40.0	0.001
1	Wash 7	20	0.01	40.0	0.001
1	Wash 8	50	0.03	45.0	0.001
1	Wash 9	50	0.05	45.0	0.002
1	Wash 10	50	0.03	48.0	0.001
1	Elution 1	500	6934.61	8.0	55.477
1	Elution 2	500	5400.09	7.0	37.801
1	Elution 3	500	2730.68	5.5	15.019
1	Elution 4	500	1537.56	9.5	14.607
1				**TOTAL µg eluted**	**122.9**
1				**% Recovery**	**78.0**
2	Lysed RBC supernatant		65.20	500.0	32.598
2	Column flow through		1.69	500.0	0.846
2	Wash 1	10	0.84	20.0	0.017
2	Wash 2	10	0.19	9.5	0.002
2	Wash 3	10	0.03	11.0	0.000
2	Wash 4	10	0.01	11.5	0.000
2	Wash 5	10	0.03	9.0	0.000
2	Wash 6	10	0.01	11.5	0.000
2	Wash 7	10	0.01	12.0	0.000
2	Wash 8	20	0.01	9.0	0.000
2	Wash 9	20	0.01	11.0	0.000
2	Wash 10	20	0.00	11.5	0.000
2	Elution 1	500	365.03	2.0	0.730
2	Elution 2	500	1426.91	2.0	2.854
2	Elution 3	500	881.73	2.0	1.763
2	Elution 4	500	304.55	3.0	0.914
2				**TOTAL µg eluted**	**6.3**
2				**% Recovery**	**19.3**

**Table 3:** Amount of nHRP2 obtained from HB3 parasite pellet from two experiments

Experiment	Sample	Imidazole (mM)	HRP2 conc (ng/mL)	Volume obtained (mL)	Total nHRP2 (µg)
1	Parasite pellet lysate		3.21	8.7	0.028
1	Flow-through		0.36	50.0	0.018
1	Wash 1	10	0.12	15.0	0.002
1	Wash 2	10	0.08	10.0	0.001
1	Wash 3	10	0.02	10.0	0.000
1	Wash 4	20	0.03	5.0	0.000
1	Wash 5	20	0.02	4.0	0.000
1	Wash 6	20	0.11	5.0	0.001
1	Wash 7	50	0.14	4.5	0.001
1	Wash 8	50	0.12	2.5	0.000
1	Wash 9	50	0.05	4.0	0.000
1	Elution 1	500	84.46	1.5	0.127
1	Elution 2	500	70.03	1.5	0.105
1	Elution 3	500	31.80	1.5	0.048
1	Elution 4	1000	19.03	4.5	0.086
1				**TOTAL µg eluted**	**0.37**
1				**% Recovery**	**1306.33**
2	Elution 1	500	1651.23	1.5	2.477
2	Elution 2	500	722.49	1.5	1.084
2	Elution 3	500	187.24	1.5	0.281
2	Elution 4	500	89.64	4.5	0.403
2				**TOTAL µg eluted**	**4.2**

For storage, elutes containing purified nHRP2 were pooled, dialyzed and concentrated using an Ultra-15 centrifugal filter (Millipore, Burlington, MA, USA). The final concentration of antigen in solution was determined by comparison with a standard curve of 0.5–20 µg/mL using a Micro BCA Protein Assay Kit (Thermo Scientific, catalog# 23225) [27]. The final purified protein was stored at −80 °C until further use.

For purification of nHRP2 from iRBC, the iRBC fractions were thawed on ice and pooled. PBS with 0.1% saponin (Sigma) was added at five times the volume of the RBCs volume and incubated for one hour on ice with gentle shaking. This suspension was centrifuged for 30 min at 17,000 rpm at 4 °C. Infected RBC lysate was used to purify nHRP2 using the same protocol as described for the supernatant.

For purification of nHRP2 from whole parasite lysate, extraction buffer containing 50 mM Tris–HCl (pH 7.5) + 5 µL Triton-X-100 + 100 mL of 150 mM NaCl and 10 mM Sodium Phosphate + 5 µL Traysol (Sigma) at pH 7.4 and protease inhibitor (Roche, Penzberg, Germany) was prepared as previously described [28]. The iRBCs were then pelleted by centrifuging at 17,000 rpm for 30 min at 4 °C. The pellet was then washed three times with cold PBS to remove haemoglobulin. Extraction buffer was added to the parasite pellet at 5 times the volume of the pellet and incubated at 4 °C on ice for 30 min. The suspension was kept on ice and sonicated a total of five times for 30 s with 5-min intervals between each round of sonication until the mixture appeared homogeneous. After sonication, the lysate was centrifuged at 17,000 rpm at 4 °C for 45 min. nHRP2 was purified from the supernatant of the parasite lysate following the procedure described above.

### SDS-PAGE and Western blotting

The washes, flow-throughs, and elutes obtained from the Ni–NTA column during nHRP2 purification were diluted at a 1:1 ratio with reducing SDS sample buffer containing Tris/Glycine/SDS prepared from a 10X stock solution (BioRad, Hercules, CA) with 0.05% 2-mercaptoethanol to prepare the sample under reducing conditions. The samples were then loaded onto an SDS gradient gel (5–20%, BioRad) with Precision plus protein dual-color standard (BioRad) used as the molecular weight marker for all western blots. Gels were electrophoresed at room temperature for 1.5 to 2 h at 100 V in a Mini Protean II Electrophoresis Cell (BioRad).

After electrophoresis, the gels were soaked for 10 min in transfer buffer containing 25 mM Tris, 192 mM glycine at pH 8.3 (BioRad), and proteins were transferred to Trans-Blot nitrocellulose paper (GE Healthcare Life Sciences, Chicago, IL, USA) at 100 milliamps constant current at 4 °C for 1 h. Following transfer, the membrane was rinsed with 1X PBS (pH 7.2) and stained with Ponceau S stain. The membrane was then washed three times with PBS 1X before blocking with 5% (wt/vol) non-fat dry milk.

For the Western blot, a mouse anti-*P. falciparum* HRP2 monoclonal antibody ((MPFG-55A, ICL Labs, Portland, OR, USA) was used as the primary antibody at dilution of 1:500 in PBS 1X with 0.5% BSA and 0.05% Tween20 with an incubation time of 60 min. The blots were then washed three times with 50 mL of PBST (PBS with 0.05% Tween20) and incubated with the secondary antibody, goat anti-mouse IgG H + L alkaline phosphatase (ThermoFisher Scientific) at a dilution of 1:2000 in PBS 1X with 0.5% BSA and 0.05% Tween20 for 45 min. Following incubation with the secondary antibody, the blots were washed three times with 50 mL of PBST as before and subsequently washed with PBS 1X for 5 min. Bands were visualized using Western Blue stabilized substrate for alkaline phosphate according to the manufacturer’s protocol (Promega, Madison, WI, USA).

### Enzyme-linked immunosorbent assay (ELISA)

A 96-well ELISA plate (Immulon 2B, ThermoFisher Scientific) was coated with nHRP2 protein at 1 µg/mL in carbonate buffer containing 0.04 M sodium bicarbonate and 0.006 M sodium carbonate (pH 9.6) and incubated overnight at 4 °C. The following day, the plate was blocked with 3% non-fat milk in PBS for 1 h at 37^0^C. Serial dilutions of the mouse anti-*P. falciparum* HRP2 monoclonal antibody (MPFG-55A) were added to the plate in triplicate at dilutions of 1:100 to 1:204,800 in PBS with 0.5% bovine serum albumin and 0.05% Tween20 and allowed to incubate for 1 h at 37 °C. Following incubation with the primary antibody, the plate was washed three times with PBST and incubated with goat anti-mouse antibody conjugated to horseradish peroxidase (HRPO) from (Abcam, Cambridge, UK) at 1:500 (equivalent to 2 µg/mL) for 45 min at 37 °C. Following the final three washes with PBST, bound antibodies were detected using TMB substrate (KPL/SeraCare, Milford, MA, USA) and absorbance values were read at 450 nm with a Molecular Devices SpectraMAX spectrophotometer (Sunnyvale, CA, USA).

### Bead-based HRP2 detection assay

The bead-based multiplex assay for malaria antigen detection was performed as described previously [29]. Magnetic microbeads (xMAP, Luminex Corp., Austin, TX, USA) were covalently bound to HRP2 capture antibodies (MPFG-55A, ICL Labs, Portland, OR, USA) by the Luminex antibody coupling kit according to manufacturer’s instructions at a concentration of 20 µg/mL. Detection antibodies were also prepared in advance by biotinylation using the EZ-link Micro Sulfo-NHS-Biotinylation Kit (ThermoFisher Scientific) according to the manufacturer’s instructions. The final prepared dilution of detection antibodies was 1.0 mg/mL for anti-HRP2 (1:1 antibody mixture of MPFG-55A and MPFM-55A, ICL Labs). Conjugated beads and prepared detection antibodies were stored at 4 °C until use in the immunoassay.

All assay reagents were diluted in buffer containing PBS pH 7.2, 0.05% Tween20, 0.5% BSA, 0.02% sodium azide. For all wash steps, the assay plate was affixed to a handheld magnet (LuminexCorp, Austin, TX, USA), and gently tapped for 2 min to allow bead magnetization before the evacuation of liquid and washing with 100 µL PBS, 0.05% Tween20. The capture beads were combined in dilution buffer and pipetted onto a BioPlex Pro 96-well assay plate (BioRad) at a quantity of approximately 800 beads/region. Plates were washed twice, and 50 µL of controls or samples were pipetted into appropriate wells. Following a 90-min gentle shaking at room temperature protected from light, plates were washed three times. Detection antibodies were prepared in dilution buffer at a concentration of 1:500 and 50 µL added to each well for a 45-min incubation. After three washes, 50 µL of streptavidin–phycoerythrin at 1:200 (Invitrogen—ThermoFisher Scientific) was added for a 30-min incubation. Plates were washed three times, and 50 µL dilution buffer was added to each well for a 30-min incubation. Plates were washed once and beads resuspended in 100 µL PBS. After brief shaking, plates were read on a MAGPIX machine (LuminexCorp) with a target of 50 beads per region. The median fluorescence intensity (MFI) value is generated for all beads collected for each region by assay well and subtracting the assay signal from wells with dilution buffer blank provides an MFI-background (MFI-bg) value used for analyses. To extrapolate from assay signal to antigen concentration, Type B recombinant HRP2 (rHRP2) was kindly provided by MicroCoat (Starnberger See, Germany) and standard curves were prepared to create a regression equation.

### Assessment of reactivity of RDTs to purified nHRP2 from HB3 culture strain

The malaria RDTs selected for this study were Carestart™ Malaria Pf (HRP2) Ag RDT (Access Bio, Inc., RMOM-02571, Lot MO19F68) and Carestart™ Malaria Pf (HRP2/pLDH) Ag RDT (Access Bio, Inc., RMPM-02571, Lot MP19F61). Six tenfold serial dilutions (in parasite-negative blood) starting at a dilution of 2060 ng/mL were prepared for the native HRP2 and recombinant HRP2 (ICL Labs). Each dilution was then tested on RDTs following manufacturer instructions by pipetting 5 µL sample and two drops of manufacturer-provided buffer to each RDT cassette. Band intensity read with a score of 0–4 was estimated for all tests. A score of 0 indicates no band was present and while scores 1 to 4 indicate positive tests with increasing band intensity. In addition to assessing RDT reactivity to the purified nHRP2, dilutions of *P. falciparum* culture strains 3D7 and US06F Nigeria XII were prepared from cultures with known parasite densities. Eight serial dilutions were prepared for 3D7 and Nigeria XII culture samples ranging between 1000 and 6.25 parasites/µL. Sample concentrations used for RDT readings are displayed in ng/mL for nHRP2 and recombinant HRP2 while parasites per µL (p/µL) concentrations are displayed for 3D7 and Nigeria XII samples.

## Results

### Binding of anti-HRP2 antibody to nHRP2 by Western blot and ELISA

**Fig. 1:**
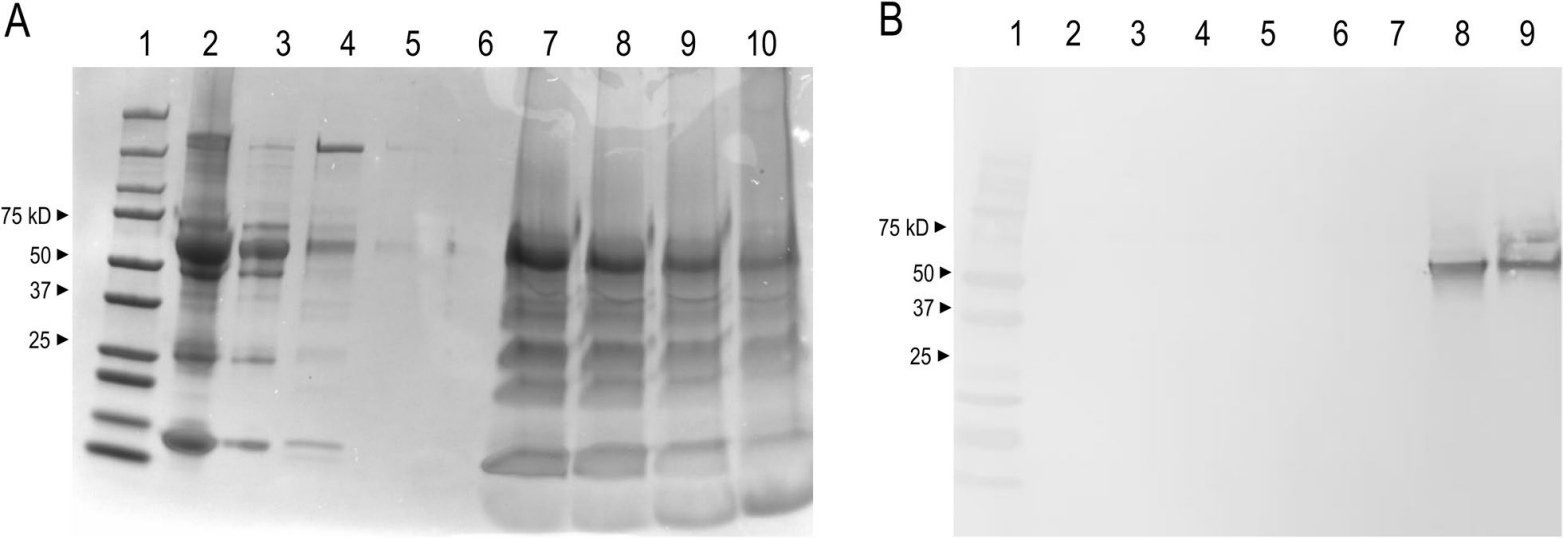
Biochemical characterization of *Plasmodium falciparum* HRP2 from HB3 culture supernatant, infected erythrocytes, and parasite lysates.** A** Coomassie-stained SDS-PAGE gel of the purified HRP2 from HB3 culture. A molecular weight ladder is shown in lane 1. The samples in each lane are as follows: 2—unprocessed supernatant, 3—column flow-through, 4—wash one, 5—wash five, 6—wash nine, 7—elute one, 8—elute two, 9—elute three, and 10—elute four. **B** Western blot of the purified HRP2 protein from the supernatant of HB3 culture. A molecular weight ladder is shown in lane 1. The samples in each lane are as follows: 2—unprocessed supernatant, 3—column flow-through, 4—wash one, 5—wash six, 6—wash seven, 7—wash nine, 8—elute one, and 9—elute four

Purified nHRP2 from HB3 culture supernatant was evaluated by SDS-PAGE. Coomassie stain revealed bands of approximately 60 kDa (Fig. 1A), and the presence of nHRP2 was confirmed via Western blotting with anti-HRP2 antibodies (Fig. 1B). Both the Coomassie gel and Western blots show the change in band intensity and clarity during the process of purification via the Ni–NTA column, going from the unprocessed cultured supernatant followed by the column flow-through, and lastly, the eluted antigen. The Coomassie gel and Western blot assay revealed a loss of many of the contaminates < 60 kDa in size between the unprocessed supernatant and the first washing, with subsequent wash steps showing no product after one wash of low imidazole. The contaminants > 60 kDa in size were lost during the washing steps resulting in an intense band visible at approximately 60 kDa in both the Coomassie gel and Western Blot. Coomassie gels and Western blots for the iRBC and parasite pellet fractions displayed similar changes in band intensity and clarity during the purification process (Additional file 1).

**Fig. 2:**
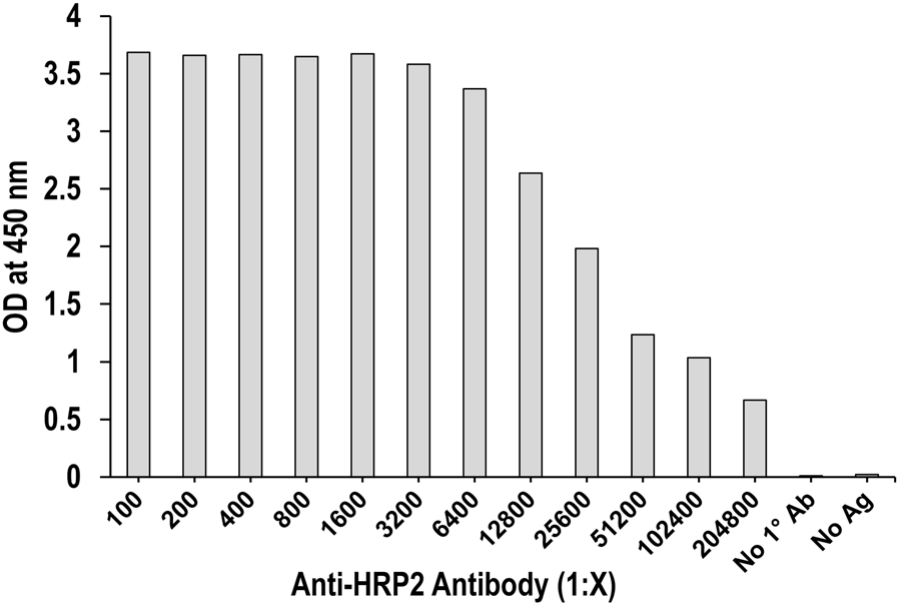
Assessment of binding of HRP2-specific mouse monoclonal antibody to nHRP2 protein by ELISA. The binding of the mouse anti-HRP2 monoclonal antibody to nHRP2 protein is shown by the mean optical density (OD) at the concentration of the monoclonal antibody indicated. Wells containing no primary antibody (no 1° ab), and no antigen (ag) were included as controls

ELISA was used to further assess the reactivity of anti-HRP2 monoclonal antibody (mAb) to the purified nHRP2. The maximum optical density (OD) signal was observed when anti-HRP2 mAb was diluted between 1:100 and 1:3200, after which the signal decreased as the mAb dilutions increased to 1:204,800 (Fig. 2).

### Quantification of nHRP2 obtained from culture supernatant, iRBCs and parasite pellet

Each fraction collected during the Ni–NTA column purification was assessed for levels of nHRP2 to assess the efficiency of nHRP2 purification, loss of antigen during the washing steps, and absolute elution of antigen from the column. Two independent column purification experiments (Exp 1 and Exp 2) were performed under slightly different wash and elution conditions to purify nHRP2 obtained from the HB3 culture supernatant (Table 1), iRBCs (Table 2), and parasite pellet (Table 3). For both experiments, high concentrations of nHRP2 were detected in the harvested culture supernatant: 40.9 ng/mL in Exp 1 and 9.6 ng/mL in Exp 2. The Ni–NTA column retained the nHRP2 antigen well, and most nHRP2 from the culture supernatant was bound by the column: 97.9% for Exp 1 and 98.8% for Exp 2. Additionally, most nHRP2 was retained from the culture supernatant during the column wash steps with the low concentrations of imidazole: 99.9% for Exp 1 and 99.9% for Exp 2. Elution with 500 or 1000 mM dissociated the nHRP2 from the Ni–NTA column and subsequent elution fractions saw decreasing amounts of nHRP2 dissociating from the column. The total amount of nHRP2 obtained from the culture supernatant elution fractions was 149.6 µg in Exp 1 and 13.6 µg in Exp 2, for a complete amount of 163.2 µg from two separate preparations.

Higher amounts of nHRP2 were seen from the lysed iRBC component of HB3 cultures: 87.6 ng/mL in Exp 1 and 65.2 ng/mL in Exp 2. As with the culture supernatants, a high proportion (> 97%) of nHRP2 from lysed iRBCs was bound to the Ni–NTA column and very little (< 0.1%) of Ni–NTA was lost during the low concentration imidazole washes. The total amount of nHRP2 obtained from the iRBC elution fractions was 122.9 µg in Exp 1 and 6.3 µg in Exp 2, for a total amount of 129.2 µg from two separate preparations.

A small amount of nHRP2 was also found in the parasite pellet obtained from the HB3 cultures but this amount was much lower in comparison to the elution fractions obtained from the culture supernatant and iRBCs. Less than 1 µg total nHRP2 was found in the parasite pellet from Exp 1 (0.4 µg), and 4.2 µg was obtained from the parasite pellet in Exp 2, for a complete amount of 4.6 µg from two separate preparations. As so little nHRP2 was found in the parasite pellet in Exp 1, only the elution fractions were quantified for Exp 2.

Among the culture supernatant, iRBC and parasite pellet fractions from two pooled collections of HB3 culture and Ni–NTA column purifications, a total yield of 296.97 µg nHRP2 was obtained from 6152.2 ml of culture materials. By volume of the culture component, the average amount of nHRP2 in each was quite similar: supernatant at 0.035 µg/mL, iRBC at 0.041 µg/mL, and parasite pellet at 0.043 µg/mL.

### Detection of purified nHRP2 by RDTs

**Fig. 3:**
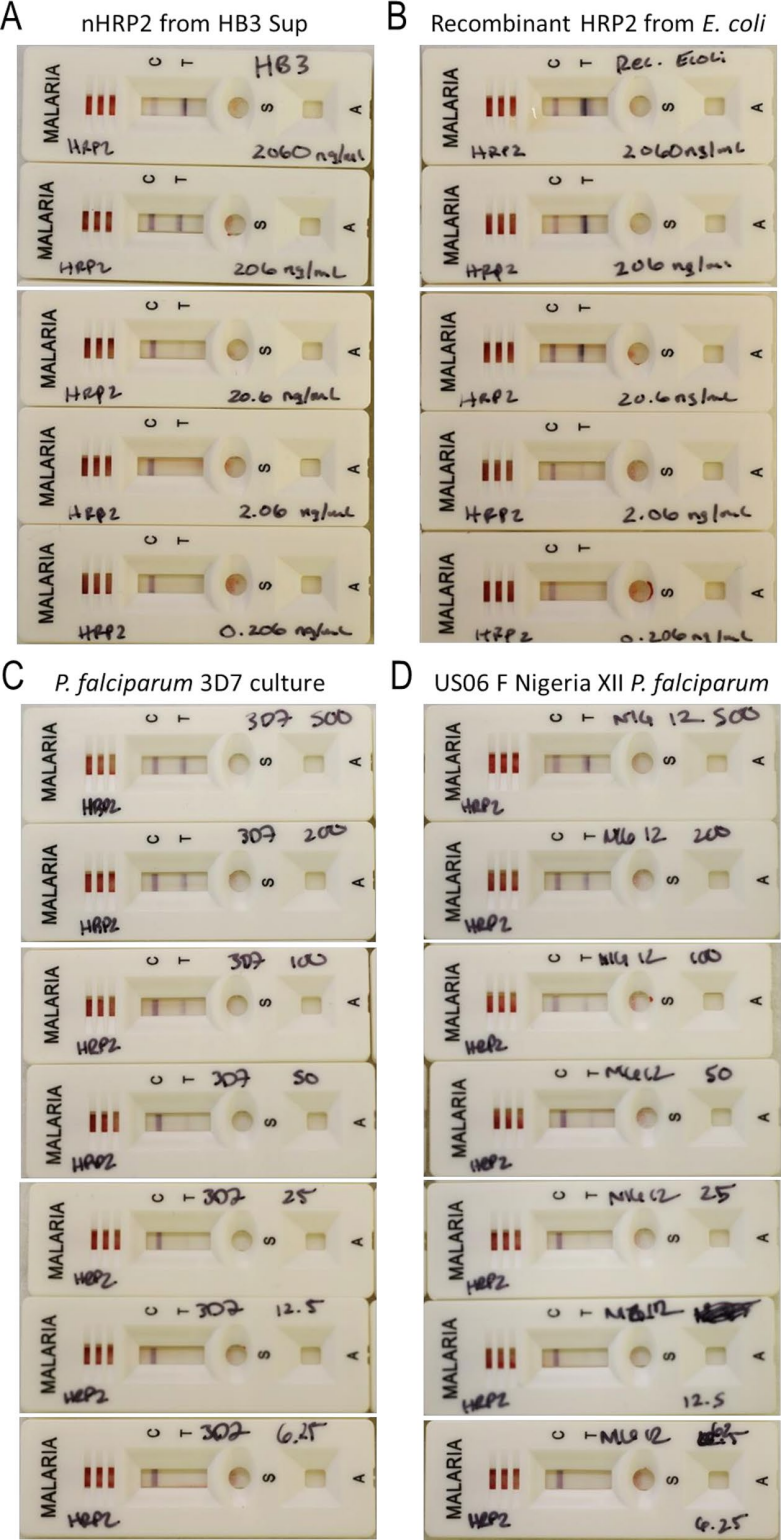
Comparison Carestart Pf HRP2 RDT band intensity between nHRP2, rHRP2, and *Plasmodium falciparum* culture samples. **A** Titration of nHRP3 from HB3 supernatant from 2060 to 0.206 ng/mL. **B** Titration of recombinant HRP2 from 2060 to 0.206 ng/mL. **C** Titration of whole parasite lysate *P. falciparum* 3D7 culture from 500 to 6.25 parasites/µL. **D** Titration of US06 F Nigeria XII *P. falciparum* parasites from 500p/µL to 6.25 parasites/µL

To confirm the reactivity of nHRP2 with commercially available RDTs, band intensity at different concentrations of nHRP2 was determined on the Carestart Pf HRP2 RDT and compared against rHRP2 and two culture strain aliquots (Table 4, Fig. 3). Both nHRP2 and rHRP2 were applied to the RDT at tenfold serial dilutions of the same concentrations. nHRP2 and rHRP2 both produced a band intensity of 4 at the highest concentration tested (2060 ng/mL) but the band intensity of nHRP2 titrated off more quickly than rHRP2. At a concentration of 2.06 ng/mL, nHRP2 did not produce a positive RDT result whereas a band could still be seen for the rHRP2 at this same concentration. For the *P. falciparum* culture strains 3D7 and US06 F Nigeria XII culture preparations (Fig. 3C and D, Table 4), band signal was lost between 12.5 and 6.25 parasites/µL of the cultured parasite preparations.

**Table 4:** Band intensity of Carestart Pf HRP2 and HRP2/pLDH RDTs following incubation with varying concentrations of nHRP2, rHRP2, and *Plasmodium falciparum* culture samples

Sample type	Sample conc	Carestart™ Malaria Pf (HRP2) Ag RDT		Carestart™ Malaria Pf (HRP2/pLDH) Ag RDT	
		Control	Line 1	Control	Line 1
nHRP2 (derived from HB3 supernatant)	2060 ng/mL	3	4	4	4
	206 ng/mL	4	3	4	3
	20.6 ng/mL	4	2	4	2
	2.06 ng/mL	4	0	4	0
	0.206 ng/mL	4	0	4	0
	0.0206 ng/mL	N/A	N/A	4	0
rHRP2	2060 ng/mL	4	4	4	4
	206 ng/mL	4	4	4	4
	20.6 ng/mL	4	4	4	3
	2.06 ng/mL	4	2	4	2
	0.206 ng/mL	4	0	4	0
	0.0206 ng/mL	N/A	N/A	4	0
Pf 3D7 culture	1000 p/µL	N/A	N/A	4	4
	500 p/µL	4	4	4	4
	200 p/µL	4	4	4	3
	100 p/µL	4	3	4	2
	50 p/µL	4	2	4	2
	25 p/µL	4	2	4	1
	12.5 p/µL	4	1	4	0
	6.25 p/µL	4	0	4	0
US06 F Nigeria XII Culture	1000 p/µL	N/A	N/A	4	4
	500 p/µL	4	4	4	4
	200 p/µL	4	4	4	3
	100 p/µL	4	3	4	2
	50 p/µL	4	2	4	1
	25 p/µL	4	2	4	1
	12.5 p/µL	4	1	4	0
	6.25 p/µL	4	0	4	0

The band intensity of nHRP2 was also compared by using the dual antigen Carestart Pf HRP2/pLDH RDT (Additional file 2, Table 4). A similar titration pattern was observed with the Carestart Pf HRP2 RDT with the rHRP2 providing a slightly higher band intensity signal than nHRP2 and able to titrate out one additional dilution point. The observed loss of positive signal for the HRP2 band occurred at 1 dilution lower for the cultured samples between 25 and 12.5 cultured parasites/µL.

## Discussion

As of 2019, more than 400 million malaria RDTs are sold annually, with most RDTs relying on the detection of *P. falciparum* HRP2 [8, 30]. There are several factors that can affect the performance of RDTs including product design, storage, transport conditions, operator errors, and parasite density within the patient sample [31–34]. As a result, the WHO Global Malaria Programme, Foundation for Innovative New Diagnostics (FIND), and the US Centers for Disease Control and Prevention have issued methods for the product testing of malaria RDTs [34], which describes the use of a specimen bank that consists of recombinant antigens, culture-derived parasites, wild-type parasites, and parasite negative blood samples for use as a reference during product testing of new and existing RDTs to ensure quality standards are met. Purified nHRP2 could also be added to the RDT quality control specimen bank established by WHO, FIND, and CDC and would allow for additional evaluation criteria for HRP2-based RDTs and help fill the gap left between the use of recombinant antigen and culture-derived parasite samples. Additionally, nHRP2 could also be utilized in laboratory research settings as the native form of this antigen that would not be subject to translational differences from recombinant production in a bacterial or other eukaryotic cell system.

This report describes the process for purification of nHRP2 protein from the HB3 *P. falciparum* culture strain, with an absence of the *pfhrp3* gene*,* making this strain an excellent source of only HRP2 and not the similar homologue HRP3. Similar efforts could also be performed with culture-adapted strains with differing gene sizes for the *pfhrp2* if isolation of different forms of this antigen is desired. In this report, the isolation of HRP2 and not HRP3 was verified by the presence of a single band in both the Coomassie stain and the Western blot at approximately 60kD, likely due to dimerization of the purified nHRP2. Although nickel-metal chelate chromatography was used here for nHRP2 purification, previous reports have described the use of zinc chelate affinity chromatography for purification of HRP2 from culture supernatant and extracts of parasitized cells [35]. Previous groups have concentrated or purified nHRP2 by a Ni–NTA protocol as well [36, 37], but did not attempt to extract large quantities of the antigen.

Characterization of nHRP2 isolated from the HB3 culture strain by SDS-PAGE and Western blot revealed the presence of an approximate 60 kDa protein that bound to mouse anti-*P. falciparum* HRP-2 monoclonal antibody, indicating successful purification of nHRP2 from culture supernatant, infected erythrocytes and whole parasite lysate. Furthermore, commercially available mouse anti-HRP2 monoclonal antibodies to nHRP2 produced stable optical density readings between dilutions of 1:100 and 1:3,200 by ELISA with signal titrating past a 1:200,000 dilution, showing the ability of nHRP2 specifically isolated from the HB3 culture strain as being well recognized by monoclonal antibodies. Due to the global variability of the *pfhrp2* gene [20, 38–42], with gene length ranging between 600–960 base pairs due to differences in the number of repeat regions within each isolate, future experiments will need to assess reactivity among other historic culture strains of *P. falciparum* that have more recently been adapted to culture and have been confirmed as *pfhrp2* + */pfhrp3*−.

Quantification of purified nHRP2 yield from HB3 culture supernatant, iRBCs, and whole parasite lysate was conducted via bead-based immunoassay and revealed that both culture supernatant and iRBC lysate were practical sources of large quantities of this antigen. Though nHRP2 was also found in the parasite pellet, such a small volume of pellet is available that it would not be pragmatic to consider this as a source for nHRP2 as well. Between two experiments, pooling the culture supernatants and iRBCs, and purifying the antigen produced a total yield of 292 µg nHRP2 which was able to be lyophilized, frozen, and reconstituted as needed for further assays. Pooling of higher parasite density cultures could provide even greater amounts of antigen, and further refining of culture and parasite growth conditions could assist in purifying milligram quantities of nHRP2.

Purified nHRP2 could be a valuable tool for the quality assessment of new and existing RDTs prior to their use in clinical settings, and the ability of Carestart Pf HRP2 and HRP2/pLDH RDTs to detect purified nHR2 was confirmed here. Both Carestart Pf RDTs detected nHRP2 when applied at concentrations between 20.6 and 2060 ng/mL. However, both Carestart Pf RDTs were slightly less reactive to nHRP2 than for rHRP2. This difference may be due to a different number of repeating epitopes between the HRP2 antigen produced by the HB3 parasite strain and the synthesized rHRP2, or in potential differences in folding between the endogenous nHRP2 and *Escherichia coli-*produced rHRP2 proteins. The band intensity observed for the nHRP2 dilutions was equivalent to that observed for *P. falciparum* culture strains 3D7 and US06 F Nigeria XII between 12.5 and 1000 parasites/µL, which suggests nHRP2 can be used alongside these established culture-derived parasite samples which are currently included in the WHO-approved product testing specimen panel [34].

## Conclusions

Due to the critical nature of HRP2-RDTs, robust and accurate methods for RDT evaluation and quality control are of extreme importance. Although the existing RDT quality control specimen bank established by WHO, FIND and CDC includes recombinant antigens, culture-derived parasites, wild-type parasites, and parasite negative blood samples, purified native HRP2 has not yet been available for use as quality control material. These results establish that not only is it possible to extract microgram quantities of the native HRP2 antigen from HB3 culture but that native HRP2 is recognized by existing monoclonal antibody lines. Purified nHRP2 has potential applications as a standard for both laboratory purposes as well as point-of-care RDTs.

## Supplementary Information

**Additional file 1: Figure S1.** Full uncropped western blots from purification process.

**Additional file 2: Figure S2.** Comparison of Band Intensity of Carestart Pf HRP2/pLDH RDT between nHRP2 and established controls.

## Data Availability

The datasets used and/or analyzed during the current study are available from the corresponding author on reasonable request.

## Abbreviations

BCA: Bicinchoninic acid
CDC: Centers for Disease Control and Prevention
ELISA: Enzyme-linked immunosorbent assay
HRP1: Histidine-rich protein-1, also called PfKAHRP
HRP2: Histidine-rich protein-2
HRP3: Histidine-rich protein-3
HRPO: Horseradish peroxidase
iRBCs: Infected red blood cells
mAb: Monoclonal antibody
nHRP2: Purified native HRP2
Ni–NTA: Nickel nitrilotriacetic acid
PBS: Phosphate buffered saline
PBST: PBS with 0.05% Tween20
PfHRP2: *P. falciparum* HRP2, used interchangeably with HRP2
PfHRP2: *P. falciparum* HRP3, used interchangeably with HRP3
PfKAHRP: *P. falciparum* Knob-associated histidine-rich protein, also called HRP1
RDTs: Rapid diagnostic tests
rHRP2: Recombinant HRP2
rpm: Revolutions per minute
SDS-PAGE: Sodium dodecyl sulfate–polyacrylamide gel electrophoresis
TMB: 3,3′,5,5′-Tetramethylbenzidine
