# Supplementary material for: Purification of native histidine-rich protein 2 (nHRP2) from Plasmodium falciparum culture supernatant, infected RBCs, and parasite lysate
Source: Malar J. 2021 Oct 17;20:405. doi: 10.1186/s12936-021-03946-1 (PMC8522059; doi:10.1186/s12936-021-03946-1)
Supplement: Supplementary file 1 — Additional file 1: Figure S1. Full uncropped western blots from purification process. [file 12936_2021_3946_MOESM1_ESM.docx]

**Additional Files**


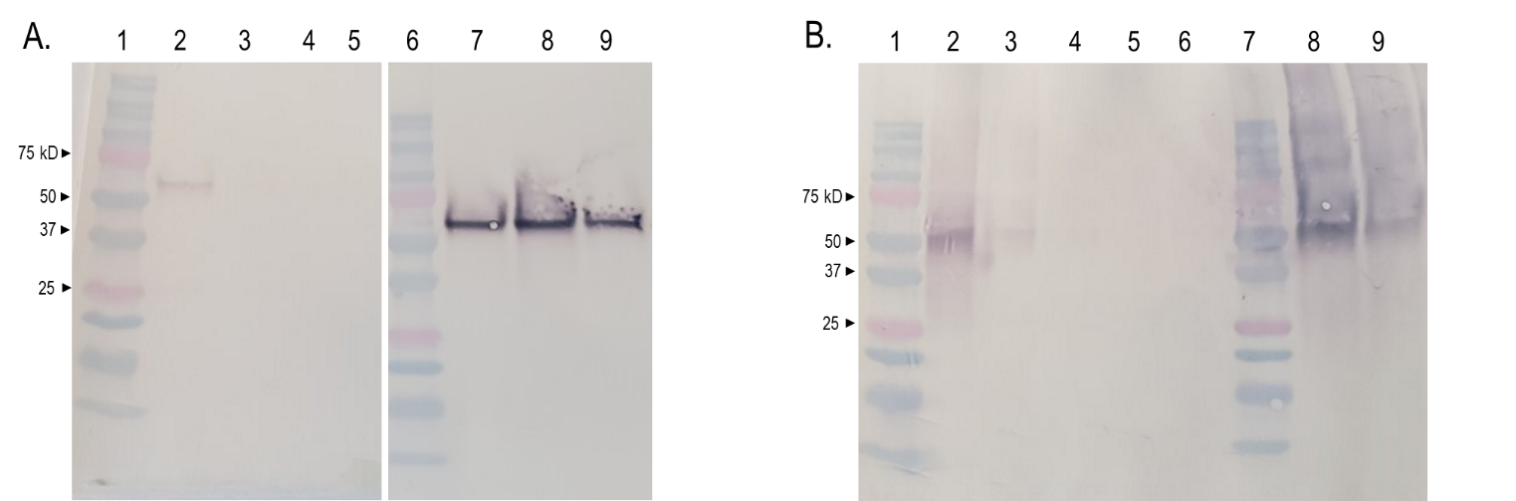


**Additional File 1.** Full Uncropped Western Blots from Purification Process. Western blot of the purified *Pf*HRP2 protein from the infected red blood cells (A) and whole parasite lysate (b) from *Pf*HB3 culture. For both Western blots, the purified HRP2 protein appears as a band of ~60 kDa. A molecular weight ladder is shown in lane 1. The samples in each lane for the iRBC Western blot is as follows: Lane 2 – unprocessed iRBCs, Lane 3 – column flow-through, Lane 4 – wash 1, Lane 5 – wash 10, Lane 6 – molecular weight ladder, Lane 7 – elute 1, Lane 8 – elute 2, and Lane 9 – elute 3. The samples in each lane for the whole parasite lysate Western blot is as follows: Lane 2 – unprocessed whole parasite lysate, Lane 3 – column flow-through, Lane 4 – wash 1, Lane 5 – wash 4, Lane 6 – wash 7, Lane 7 – molecular weight marker, Lane 8 – elute 1, Lane and 9 – elute 3. For both western blots, a mouse anti-HRP2 monoclonal antibody (ICL Inc.) was used as the primary antibody at dilution of 1:500. A goat anti-mouse alkaline phosphatase (ThermoFisher) was used as the secondary antibody at a dilution of 1:2,000.
