# Supplementary material for: Purification of native histidine-rich protein 2 (nHRP2) from Plasmodium falciparum culture supernatant, infected RBCs, and parasite lysate
Source: Malar J. 2021 Oct 17;20:405. doi: 10.1186/s12936-021-03946-1 (PMC8522059; doi:10.1186/s12936-021-03946-1)
Supplement: Supplementary file 2 — Additional file 2: Figure S2. Comparison of Band Intensity of Carestart Pf HRP2/pLDH RDT between nHRP2 and established controls. [file 12936_2021_3946_MOESM2_ESM.docx]

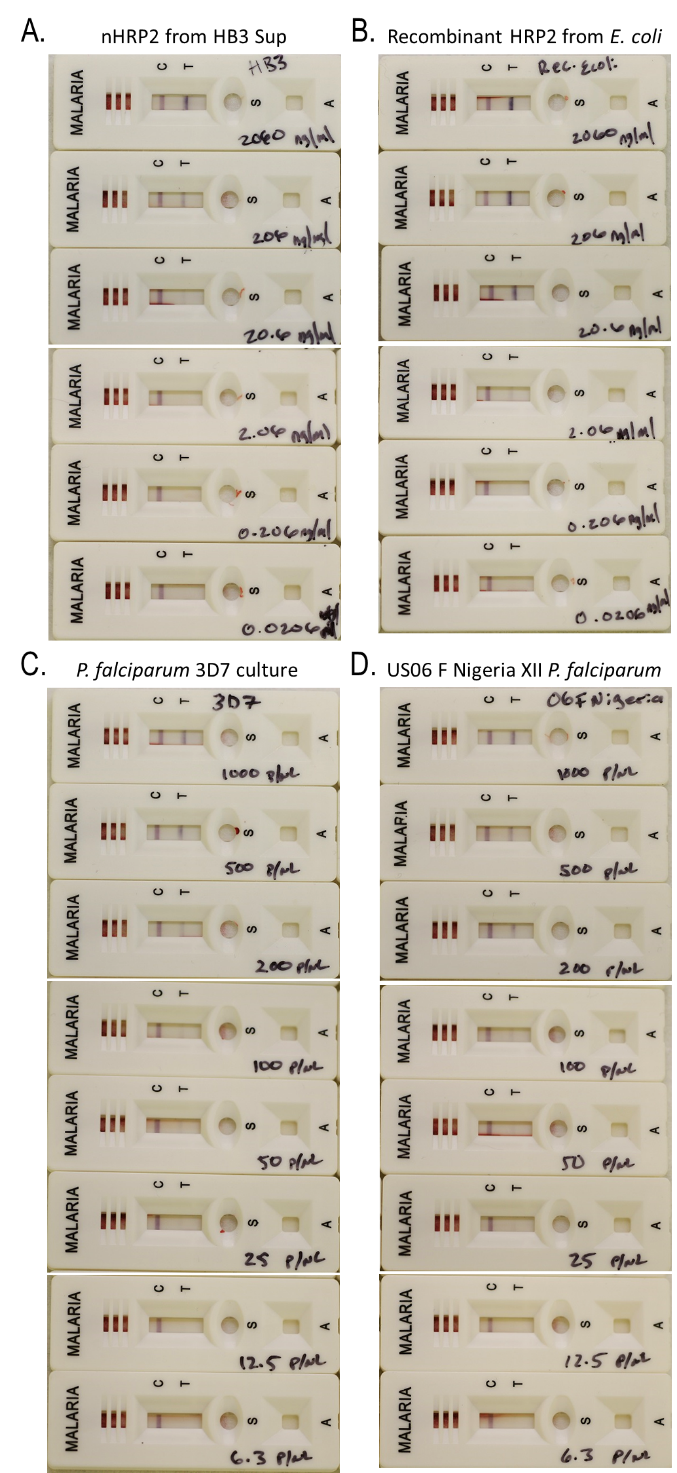


**Additional File 2. Comparison of Band Intensity of Carestart Pf HRP2/pLDH RDT between nHRP2 and established controls.** A) Titration of nHRP3 from HB3 supernatant from 2060 – 0.0206 ng/mL. B) Titration of recombinant HRP2 produced in *E. coli* (ICL Labs) from 2060-0.0206 ng/mL. C) Titration of whole parasite lysate *P. falciparum* 3D7 culture from 1000 – 6.25 parasites/µL. D) Titration of US06 F Nigeria XII *P. falciparum* parasites from 1000p/µL – 6.25 parasites/µL.
